# Structural Basis for the Regulation of Maternal Embryonic Leucine Zipper Kinase

**DOI:** 10.1371/journal.pone.0070031

**Published:** 2013-07-26

**Authors:** Lu-Sha Cao, Jue Wang, Yuling Chen, Haiteng Deng, Zhi-Xin Wang, Jia-Wei Wu

**Affiliations:** MOE Key Laboratory of Protein Sciences and Tsinghua-Peking Center for Life Sciences, School of Life Sciences, Tsinghua University, Beijing, China; National Institute for Medical Research, Medical Research Council, London, United Kingdom

## Abstract

MELK (maternal embryonic leucine zipper kinase), which is a member of the AMPK (AMP-activated protein kinase)-related kinase family, plays important roles in diverse cellular processes and has become a promising drug target for certain cancers. However, the regulatory mechanism of MELK remains elusive. Here, we report the crystal structure of a fragment of human MELK that contains the kinase domain and ubiquitin-associated (UBA) domain. The UBA domain tightly binds to the back of the kinase domain, which may contribute to the proper conformation and activity of the kinase domain. Interestingly, the activation segment in the kinase domain displays a unique conformation that contains an intramolecular disulfide bond. The structural and biochemical analyses unravel the molecular mechanisms for the autophosphorylation/activation of MELK and the dependence of its catalytic activity on reducing agents. Thus, our results may provide the basis for designing specific MELK inhibitors for cancer treatment.

## Introduction

Maternal embryonic leucine zipper kinase (MELK), which is also known as murine protein serine/threonine kinase 38 (MPK38) and pEg3 kinase, is a member of the AMP-activated protein kinase (AMPK)-related kinase family [1]–[4]. MELK is a cell cycle-dependent protein kinase that is involved in the regulation of various biological processes, including cell proliferation [5]–[8], spliceosome assembly [9], hematopoiesis [10], stem cell self-renewal [11] and apoptosis [12], [13]. Interestingly, the expression level of MELK is dramatically increased in multiple cancer tissues, which may be relevant for establishing and/or maintaining certain types of tumor [14], [15]. The level of MELK is also associated with the malignancy grade in brain tumors and the poor prognosis in breast and brain cancer patients [16]–[18]. Thus, MELK is a promising drug target for cancer treatment and an important prognosis marker for some cancers.

**Figure 1:**
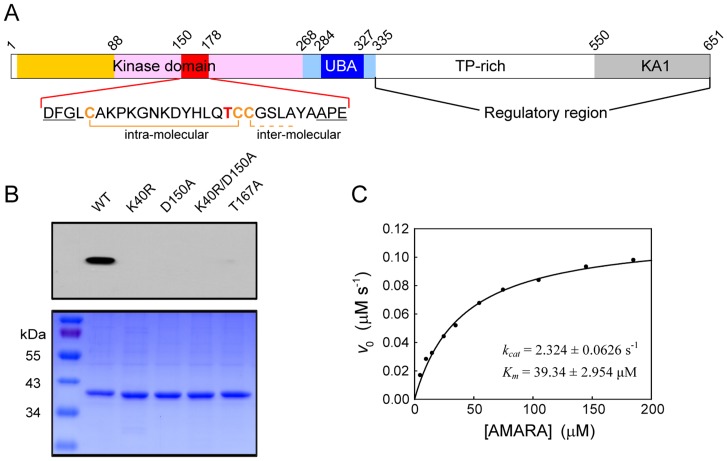
Autophosphorylation of MELK KD-UBA. (A) Schematic diagram of MELK. The domains are colored as follows: kinase domain (N-lobe, orange; C-lobe, pink), UBA domain (blue), linker regions flanking UBA (slate blue) and KA1 domain (gray). The sequence of the activation segment (red) is provided, with the Thr residue required for kinase activity and the Cys residues involved in the formation of the disulfide bonds highlighted in bold. Residue numbers refer to human MELK (Swiss-Prot entry Q14680). (B) Phosphorylation state of Thr167 in the activation segment. Upper row: broad-spectrum phospho-Thr antibody detection; lower row: Coomassie blue staining. (C) Dependence of the initial velocity of the MELK KD-UBA-catalyzed reaction on the concentration of the AMARA peptide. The assays were carried out in a standard buffer containing 50 nM wildtype MELK KD-UBA, 10 mM DTT and the indicated amounts of AMARA. The solid line represents the best fit to the Michaelis-Menten equation with *k_cat_* and *K_m_* values of 2.324±0.0626 s^−1^ and 39.34±2.954 µM, respectively.

MELK is highly conserved among species ranging from human to *C. elegans* [1], [3], [4], [7], [19] and comprises an N-terminal Ser/Thr kinase domain and an adjacent ubiquitin-associated (UBA) domain (Figure 1A). The C-terminal regulatory region contains a TP-rich region and a kinase-associated 1 (KA1) domain, and was suggested to autoinhibit the kinase activity of MELK [20]. The TP-rich region contains multiple phosphorylation sites [20], and phosphorylation of a specific Thr residue within this region is required for the inhibition of spliceosome assembly by MELK [9]. The KA1 domain contributes to the membrane association of some AMPK-related kinases [21].

In the human genome, the only protein kinase family that includes the UBA domain is the AMPK-related kinase family [22]. The canonical UBA domains are known to associate with ubiquitin and thereby prevent ubiquitin-dependent protein degradation [23]; however, the UBA domains in the AMPK-related kinases adopt a noncanonical conformation and lack significant ubiquitin-binding activity [24], [25]. The microtubule affinity regulating kinases (MARK1-4) are the most studied AMPK-related kinases, which were first identified by their ability to phosphorylate tau and related microtubule-associated proteins [26]. The UBA domains of MARKs were thought to be required for the phosphorylation and activation by the upstream kinase LKB1 [24]; in contrast, the structural and biochemical studies of MARKs revealed that the UBA domain directly binds to and inhibits the kinase domain [27]. The αβγ-heterotrimeric AMPK plays a central role in energy homeostasis [28]. The α-subunit of AMPK shares a similar architecture as other AMPK-related kinases, and its UBA domain is referred to as the autoinhibitory domain (AID) due to its inhibitory effect on AMPK kinase activity [29], [30]. One of the AMPK energy-sensing mechanisms involves allosteric activation upon AMP binding to the regulatory γ-subunit, and we have demonstrated that the AID is also involved in the allosteric regulation of AMPK [29], [30]. These results suggest that the UBA domains of AMPK-related kinases may play distinct roles in regulating their kinase activities. However, the role of the UBA domain in MELK remains elusive.

The activity of the AMPK-related kinase family is tightly regulated by LKB1 through the phosphorylation of a highly conserved Thr residue within the activation segment [2]. However, MELK is unique among these AMPK-related kinases in that it is not activated by LKB1 but undergoes autophosphorylation at the key Thr (Thr167 in human MELK) and additional Ser/Thr residues in the kinase, UBA and TP-rich domains [2], [20]. Once activated, MELK can regulate the aforementioned important biological processes through the phosphorylation of its protein substrates, including the cell cycle protein phosphatase CDC25B [5], [8], the proapoptotic molecule Bcl-G and apoptosis signal-regulating kinase ASK1 [12], [13], the TGF-β signal transducer Smad proteins [31], the tumor suppressor p53 [32] and the phosphoinositide-dependent kinase PDK1 [33]. Thus, the autophosphorylation/activation and regulation of the catalytic activity of MELK is crucial to its biological function.

To understand the molecular mechanism of MELK regulation, we carried out structural and biochemical studies on the MELK fragment containing the kinase and UBA domains (KD-UBA). The crystal structure of MELK KD-UBA reveals that the UBA domain tightly binds to the N-lobe of the kinase domain, and structure-based mutagenesis indicates that the UBA domain might assist in the appropriate folding of the kinase domain and/or directly regulate its catalytic activity. Intriguingly, the kinase domain adopts a conformation compatible with ATP⋅Mg^2+^ binding but not the recruitment of an exogenous substrate, which is partially due to the presence of a unique intramolecular disulfide bond within the activation segment. The structural and biochemical features of MELK provide a better understanding of the regulation of this important protein kinase and may facilitate the development of specific MELK inhibitors for cancer treatment.

## Materials and Methods

### Protein preparation

The plasmid of human MELK was kindly provided by Dr. Jean-Pierre Tassan (Université de Rennes 1). The KD-UBA fragment (residues 1–340) was subcloned into the vector pET21b (Novagen) using NdeI/XhoI restriction sites. The site-specific mutations were generated by overlap PCR and verified by DNA sequencing. The plasmids were transformed into the *E. coli* BL21(DE3) strain. Cultures were grown in standard LB medium at 37°C to an OD_600_ of approximately 0.6 and then induced with 0.2 mM isopropyl-β-d-thiogalactopyranoside (IPTG) at 20°C for 12–16 h. Cells were collected by centrifugation and lysed by sonication. The MELK proteins containing a C-terminal His_6_-tag were purified at 4°C using Ni-NTA chromatography (Qiagen) and eluted with 300 mM imidazole. The eluates were then subjected to ion exchange chromatography (Source 15Q, GE Healthcare) and eluted with a linear 0–1 M NaCl gradient. After concentration, the proteins were further purified using size exclusion chromatography (Superdex 200, GE Healthcare) and eluted with a buffer containing 10 mM Tris-HCl (pH 8.0) and 150 mM NaCl in the presence or absence of 5 mM dithiothreitol (DTT). The purified proteins were stored at −80°C and subjected to crystallization trials. Protein stocks for kinetic analysis were supplemented with glycerol at a final concentration of 20% (v/v).

### Kinetic analysis of MELK kinase activity

The enzymatic activity of MELK was spectrophotometrically determined using the synthetic AMARA peptide as substrate [34]. The spectrophotometric assay couples the production of ADP with the oxidation of NADH by pyruvate kinase and lactate dehydrogenase [35], [36]. The AMARA peptide (AMARAASAAALA) was synthesized by Zhongke Yaguang Inc., and characterized by MALDI-TOF mass spectrometry. The assay was performed at 25°C in a 1.8 ml reaction mixture containing 50 mM MOPS (pH 7.0), 100 mM NaCl, 0.1 mM EDTA, 10 mM MgCl_2_, 0.2 mM NADH, 1 mM ATP, 1.0 mM phosphoenolpyruvate, 20 units/ml lactate dehydrogenase, 15 units/ml pyruvate kinase, and varying amounts of the AMARA peptide, enzyme and/or DTT as indicated. The reactions were initiated by the addition of the AMARA peptide to the reaction mixture. The progress of the reaction was continuously monitored by measuring the formation of NAD^+^ at 340 nm on a PerkinElmer Lambda 45 spectrophotometer (PerkinElmer Life Sciences) equipped with a magnetic stirrer in the cuvette holder. The initial rates were determined from the slopes of the progress curves, and the experimental data were analyzed using a nonlinear regression analysis program. The concentrations of ADP formed in the MELK-catalyzed reaction were determined with an extinction coefficient for NADH of 6,220 M^−1^ cm^−1^ at 340 nm. The concentration of the AMARA peptide was determined by finishing the reaction under conditions of limiting peptide. The kinetic parameters were obtained by fitting the experimental data to the Michaelis-Menten equation.

### Crystallography

The crystal of the KRDA mutant of MELK KD-UBA was grown using the hanging-drop vapor diffusion method by mixing the protein (approximately 8 mg/ml) with an equal volume of reservoir solution containing 0.1 M sodium cacodylate (pH 6.5), 0.7 M sodium acetate and 2% PEG 400 at room temperature. Crystals were equilibrated in a cryoprotectant buffer containing reservoir solution supplemented with 20% ethylene glycol and flash frozen under a cold nitrogen stream at 100 K. The diffraction data sets were collected at 2.75 Å at beamline 17U of the Shanghai Synchrotron Radiation Facility (SSRF, Shanghai, China) and processed using HKL2000 [37]. The structure was solved by molecular replacement using Phaser [38] and the MARK3 KD-UBA structure (PDB ID: 3FE3) as the search model. Standard refinement was performed using Phenix [39] and Coot [40]. The data processing and refinement statistics are summarized in Table 1. The atomic coordinates and structure factors have been deposited in the PDB (http://www.pdb.org) under the accession number 4IXP.

**Table 1:** Data collection and refinement statistics.

**Data Collection** a	
Space group	*P6_4_22*
Cell dimensions	
*a, b, c* (Å)	135.45, 135.45, 153.65
α, β, γ (°)	90, 90, 120
Resolution (Å)	50.00-2.75 (2.80-2.75)^b^
Total reflections	156642
Unique reflections	22247
No. reflections used	22206
*R*_merge_ (%)	5.9 (47.3)
*I/σ* (*I*)	30.0 (4.6)
Completeness (%)	99.8 (100)
Redundancy	7.0 (7.3)
**Refinement statistics**	
Resolution (Å)	34.85-2.75 (2.87-2.75)
*R*_work_/*R*_free_ (%)	20.2/23.2 (30.97/38.73)
No. Atoms	
Protein	2719
Water	39
B-factors	
Protein	74.76
Water	57.32
Rmsd Bond lengths (Å)	0.008
Rmsd Bond Angles (°)	1.110
**Ramachandran plot statistics**	
Most favored (%)	91.1
Additionally allowed (%)	8.9
Generously allowed (%)	0.0
Disallowed (%)	0.0

a All data sets were collected from a single crystal.

b Values in the parentheses are for the highest-resolution shell.

## Results and Discussion

### Overall structure of MELK KD-UBA

To determine the regulatory mechanism of MELK, we overexpressed some truncation mutants of human MELK in *E. coli* but only obtained soluble protein of the N-terminal fragment containing the predicted kinase and UBA domains (KD-UBA, residues 1–340) (Figure 1A). The bacterially expressed KD-UBA protein was autophosphorylated (Figure 1B), which is consistent with a previous report [20]. Mass spectrometry analysis of this KD-UBA fragment demonstrated that the critical Thr167 was indeed phosphorylated (Figure S1). Accordingly, the purified MELK KD-UBA can phosphorylate the AMARA peptide, which is a widely used substrate for AMPK-related kinases (Figure 1C). These results suggest that MELK is able to undergo autophosphorylation and that its kinase activity is tightly regulated by the phosphorylation of Thr167.

To better understand the autophosphorylation mechanism, we carried out crystallization trials with the wildtype MELK KD-UBA protein in the presence or absence of the nonhydrolyzable ATP analog AMP-PNP, however, without success. In addition to Thr167,multiple autophosphorylation sites were reported, which include Thr56, Tyr163, Ser171, Ser253 and Ser336 that are present within our KD-UBA fragment [20]. We therefore speculated that the heterogeneity of the autophosphorylated MELK proteins might be the primary obstacle and generated several kinase-dead mutants through substitution of the highly conserved Thr167, Lys40 and/or Asp150. Lys40 is indispensable for ATP binding through the stabilization of the α- and β-phosphate groups, while Asp150 in the conserved DFG motif is crucial for the recognition of the ATP-bound magnesium ion. Indeed, these mutations abrogated the catalytic activity of MELK, which is consistent with their undetectable autophosphorylation (Figure 1B).

**Figure 2:**
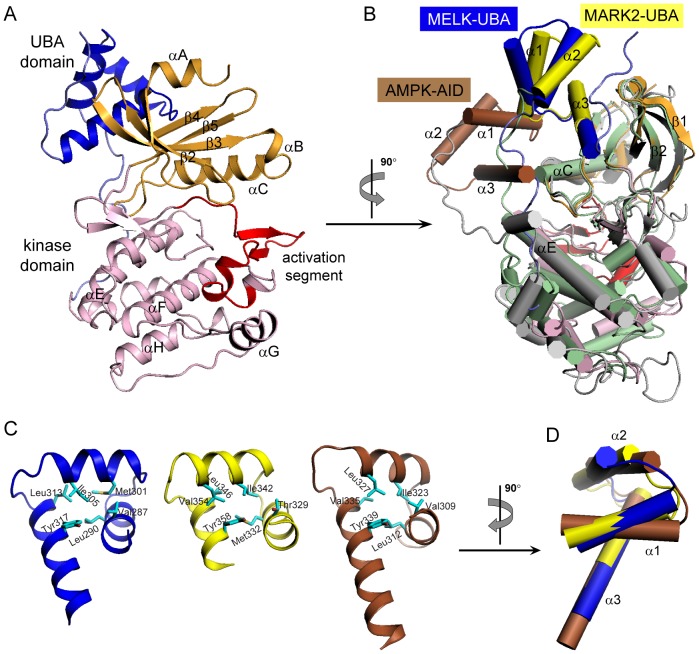
Overall Structure of MELK KD-UBA. (A) Ribbon representation of MELK KD-UBA using the identical coloring scheme as in the schematic diagram in Figure 1A. All structural representations were prepared with PyMOL (http://www.pymol.org). (B) Structural comparison of MELK KD-UBA with MARK2 KD-UBA (2WZJ) and AMPK KD-AID (3H4J) upon superposition of the N-lobes of their kinase domains. The kinase and UBA domains of MARK2 are respectively shown in light green and yellow, and the corresponding domains of AMPK KD-AID in gray and brown. (C) Conserved hydrophobic cores of MELK-UBA, MARK-UBA and AMPK-AID. The hydrophobic residues are highlighted as cyan sticks. (D) Difference in the helix arrangement of three UBA domains from MELK, MARK and AMPK upon superposition of their α3 helices.

**Figure 3:**
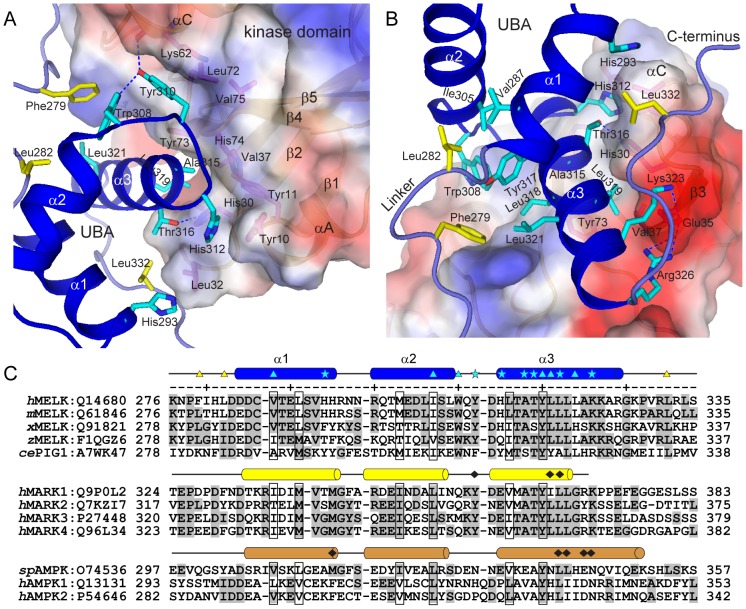
Tight interactions between the kinase domain and the UBA domain. (A and B) Hydrophobic and hydrophilic interactions at the KD-UBA interface. The kinase domain is shown in surface representation and is colored according to the electrostatic potential (positive, blue; negative, red). The residues from the UBA domain are highlighted as cyan sticks, and the residues from two linkers flanking the UBA domain and from the kinase domain are highlighted as yellow and magenta sticks, respectively. Hydrogen bonds are indicated by blue dashed lines. (C) Sequence alignment of MELK-UBAs, MARK-UBAs and AMPK-AIDs. The code following each protein name is the corresponding Swiss-Prot ID. The residues of MELK-UBA involved in the interaction with the kinase domain are indicated by cyan asterisks, and the interacting residues of MARK-UBA and AMPK-AID are indicated by black diamonds. The residues involved in the interactions between MELK-UBA and its flanking linkers are indicated by cyan and yellow triangles, respectively. *h*, human; *m*, mouse; *x*, *Xenopus*; *z*, zebrafish; *sp*, *S. pombe*.

All of these kinase-dead mutants were subjected to crystallization trials, and fortunately, the K40R/D150A double mutant yielded crystals with one molecule per asymmetric unit. The structure was determined by molecular replacement and refined to 2.75 Å resolution (Table 1). This human MELK KD-UBA structure contains residues 2–335, which fold into a kinase domain followed by a noncanonical UBA domain (Figure 2A). The kinase domain adopts the characteristic bilobal fold in which the N-lobe contains a five-stranded antiparallel β-sheet and the universally conserved αC helix and the C-lobe is mostly α-helical. Notably, the UBA domain of MELK (MELK-UBA) binds to the back of the kinase domain on the opposite face of the catalytic cleft, which is similar to that observed in MARKs but distinct from that in AMPK (Figure 2B). Although the UBA domains in the AMPK-related kinases exhibit low sequence homology, all of the determined structures display the noncanonical UBA fold that consists of helices α1-α3 [25], [27], [29], [41]–[43]. This compact conformation differs from the canonical UBA fold by the inversion of helix α3, which is stabilized by multiple van der Waals interactions involving several conserved hydrophobic residues (Figure 2C and Figure 3C). The most obvious structural deviations are mapped to helix α3, which varies in length from two to five turns. In addition, the relative orientation of the three helices in MELK-UBA is markedly distinguishable from that in MARK2-UBA and AMPK-AID (Figure 2D). The conformational diversity of these UBA domains may be important for their different biological functions in specific AMPK-related kinases.

### MELK-UBA tightly binds to the N-lobe of the kinase domain

We have demonstrated that the AID of AMPK binds to both N- and C-lobes of its kinase domain, which constrains the mobility of the prominent αC helix and thereby inhibits the kinase domain [29]. Distinctly, the UBA domains in MELK and MARKs exclusively bind to the N-lobe of the kinase domain (Figure 2B). In MELK, the aromatic side chain of Tyr310 from the UBA domain penetrates into the hydrophobic pocket formed by the methylene groups of Lys62 and two hydrophobic residues (Val75 and Leu72) from the kinase N-lobe (Figure 3A). The hydroxyl group of Tyr310 also forms two hydrogen bonds with Lys62 and Trp308. The highly conserved hydrophobic residues Leu319 and Ala315 in helix α3 of the UBA domain lean against a hydrophobic groove formed by His30, Val37, Tyr73 and His74 from the N-lobe β-sheet (Figure 3A and B). These predominantly hydrophobic interactions are largely conserved in MARKs (Figure S2).

In addition, in MELK the variant His312 at the N-terminus of helix α3 inserts its imidazole ring into a hydrophobic pocket formed by Tyr10, Tyr11, His30 and Leu32 from the kinase domain (Figure 3A). In turn, His30 hydrogen bonds to Thr316 on the UBA α3 helix, and Leu32 forms van der Waals interactions with His293 on the UBA α1 helix and Leu332 located in the loop that is C-terminal to the UBA domain. Additional polar interactions are formed between two basic residues Lys323 and Arg326 on the UBA α3 helix and the negatively charged Glu35 in the N-lobe (Figure 3B). Therefore, in MELK the UBA α1 helix and the C-terminal loop, in addition to helix α3, participate in multiple interactions with the kinase domain. Interestingly, Phe279 and Leu282 from the linker between the kinase and UBA domains of MELK interact with six hydrophobic amino acids from the UBA domain (Figure 3B). This hydrophobic surface lies on the opposite face of the MELK UBA domain from the UBA-kinase domain interface and consists of residues that are not well conserved in the MARK UBA domain. More importantly, His312 in MELK is substituted with an acidic Glu residue in the MARK-UBAs (Glu353 in human MARK2), and the acidic Glu35 in the MELK kinase domain is replaced by a basic side chain of Arg or Lys (Lys77 in human MARK2) (Figure 3C and Figure S3). Thus, most of the additional hydrophobic and hydrophilic interactions between the UBA and kinase domains of MELK are absent in MARK (Figure S2). Binding of the UBA domain to the kinase domain of MELK results in a buried surface area of more than 1600 Å^2^, which is significantly larger than that in MARK2 (approximately 1200 Å^2^). These structural analyses clearly indicate a tighter association between the kinase and UBA domains in MELK.

### Roles of the UBA Domain in MELK

**Figure 4:**
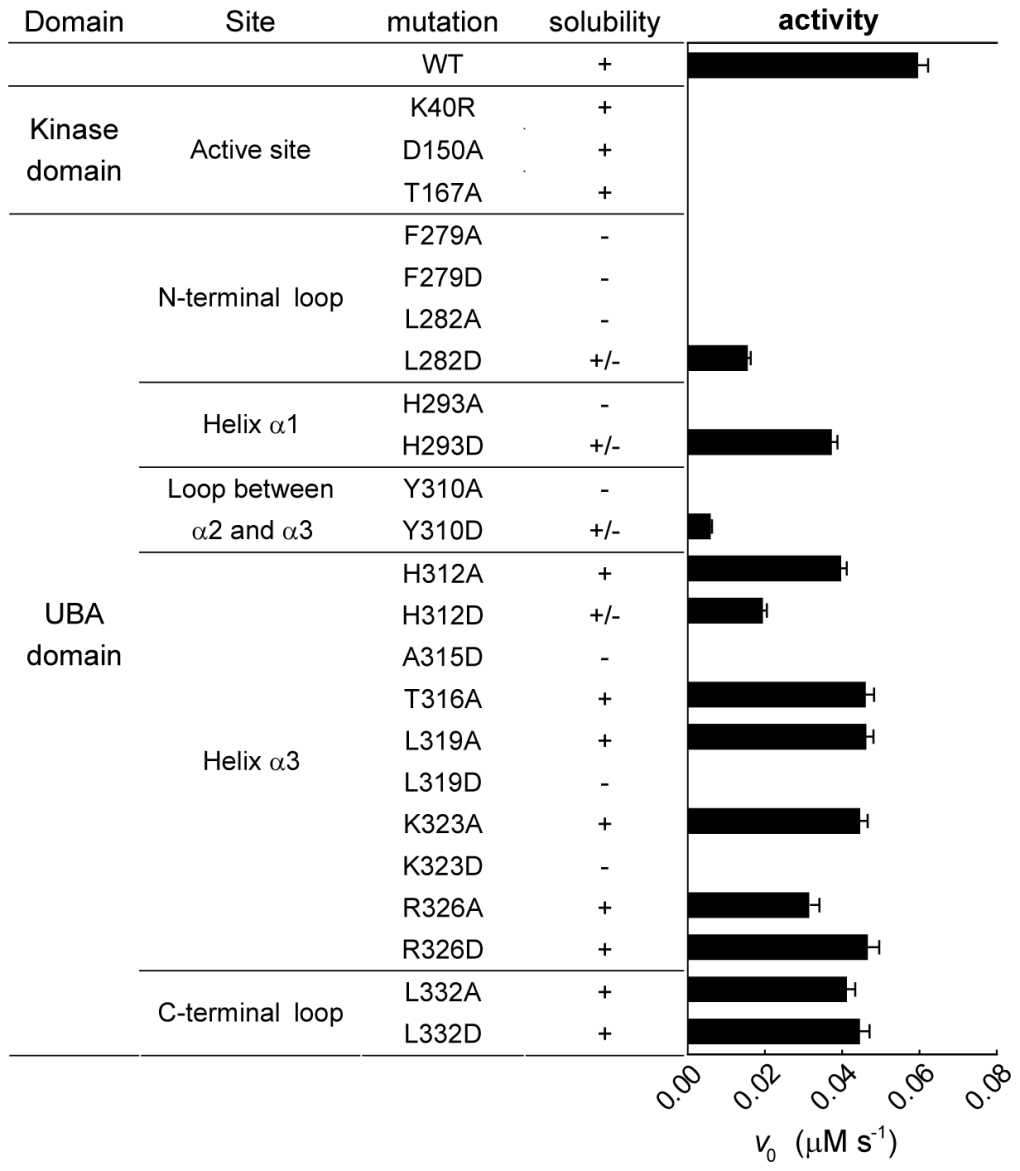
Solubility and activity of MELK KD-UBA mutants. All mutants were overexpressed in *E. coli* and the soluble proteins were purified to homogeneity as described in the Materials and Methods. The kinase assay was performed in the presence of 10 µM AMARA, 10 mM DTT and 100 nM MELK in standard kinase assay buffer (mean and s.e.m., n = 3).

To assess the importance of these interactions, we generated a series of point mutations located in the UBA domain and two flanking loops in the MELK KD-UBA fragment. Some mutations dramatically reduced the solubility of the mutant proteins when expressed in *E. coli*, which was likely caused by the expected drastic destabilizing effects (Figure 4). For instance, mutation of Phe279 in the N-terminal loop or His293, Ala315, Leu319 at the UBA-kinase domain interface would expose hydrophobic patches on the surfaces of the UBA or kinase domains (Figure 3B), resulting in the nonspecific aggregation of the mutant proteins. Considering that all efforts to generate an isolated kinase domain were unsuccessful, we speculate that the aforementioned extensive interactions might play a crucial role in the folding of the nascent polypeptides of the MELK KD-UBA fragment.

All soluble mutants were purified to homogeneity, and their effects on the catalytic function of MELK KD-UBA were examined. The folding properties of the proteins were first assessed by circular dichroism, and the results indicate that the soluble or partially soluble mutants largely fold into the native conformation (Figure S4). However, the catalytic activities of these mutants were more or less impaired compared with that of the wildtype KD-UBA (Figure 4). In particular, to largely eliminate the hydrophobic and hydrophilic interactions, we replaced Tyr310 with the negatively charged Asp (Y310D), which led to the most dramatic reduction of kinase activity (approximately 90%). The individual mutation of His293 and His312 to a charged residue (H293D and H312D) and substitution of Leu319 to the small hydrophobic Ala (L319A) yielded a modest decrease because of the diminished hydrophobic interaction. The mutants T316A, K323A and R326A, which were engineered to abrogate the hydrophilic interactions, also moderately impaired the kinase activity. These results suggest that the UBA domain may also function to maintain the appropriate conformation of the kinase domain and thereby enhance the activity of MELK by directly binding to the critical αC helix.

We next examine the role of the loops flanking the UBA domain in regulating MELK activity. Mutating Leu332 in the C-terminal loop to Ala or Asp demonstrated little effect on the solubility and activity, which is consistent with the disordered nature of the C-terminal tail in MARKs. In contrast, substitution of Phe279 and Leu282 in the N-terminal loop dramatically reduced the solubility of the resulting proteins, and the catalytic activity of the partially soluble mutant L282D was largely diminished. These results indicate that the proper conformation of the UBA domain is indispensable for its function because Leu282 interacts with three hydrophobic core residues of the UBA domain, Val287, Ile305 and Tyr317 (Figure 3B and C). Thus, we speculate that the UBA domain is essential for the catalytic activity of MELK in that it may contribute to the protein folding and proper conformation of the kinase domain.

### The active site in the unphosphorylated MELK

**Figure 5:**
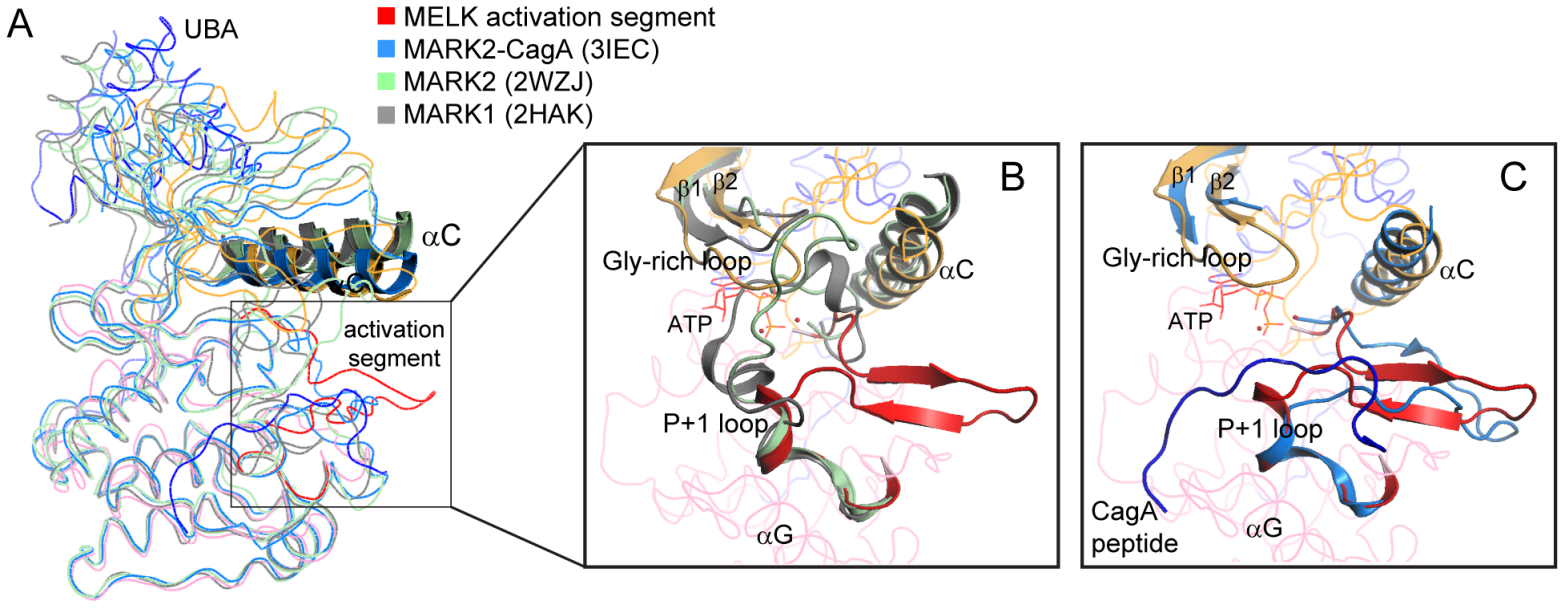
Conformational differences between the activation segments in MELK and MARK KD-UBA structures. (A) Superposition of four KD-UBA structures with ordered activation segments. The MELK KD-UBA structure is colored as in Figure 2A, the MARK2-CagA complex (3IEC) in marine blue, the double mutant MARK2 (2WZJ) in light green, and the wildtype MARK1 (2HAK) in gray. For clarity, only the αC helices are depicted in cartoon. (B) Close-up view of the activation segments of MELK, MARK2 (2WZJ) and MARK1 (2HAK). The molecules are colored as in panel A. For clarity, the activation segment, helix αC and Gly-rich loop in MELK are highlighted, and only these elements in MARK1 and 2 are shown. The ATP molecule adapted from PKA (1ATP) is shown as red lines. (C) Close-up view of the activation segments of MELK and the MARK2-CagA complex (3IEC). For the MARK2-CagA complex, only the activation segment, helix αC, Gly-rich loop and the CagA peptide (blue) are shown.

The mutation of two crucial residues required for ATP and magnesium binding (K40R/D150A) results in an unphosphorylated, inactive kinase (Figure 1B). Notably, although the activation segment between and including the DFG and APE motifs is disordered in most inactive kinase structures [44], [45], this segment (residues 150–178) in our unphosphorylated MELK structure is traceable. The activation segments in three structures of MARK1 and MARK2 are also ordered [27], [42], [43]. Comparison of these KD-UBA structures, which are superposed using their kinase C-lobes, reveals substantial conformational differences (Figure 5). The structures of the wildtype MARK1 KD-UBA (PDB ID: 2HAK) and the K82R/T208E double mutant MARK2 (2WZJ) were thought to represent the inactive conformation, whereas the structure of the wildtype MARK2 KD-UBA fragment in complex with an inhibitory CagA peptide (3IEC) adopts the active, substrate-bound conformation [42], [43]. Intriguingly, as indicated by the different orientations of the prominent αC helix, the kinase domain in our unphosphorylated MELK KD-UBA structure adopts a relatively closed conformation that is similar to the active MARK2-CagA complex (Figure 5A). The activation segments in the inactive MARK structures are folded into the catalytic cleft between the N- and C-lobes, which hinder the binding of an ATP molecule; in contrast, the activation segment in our unphosphorylated MELK KD-UBA protrudes from the cleft, which allows ATP⋅Mg^2+^ to bind, and the ordered Gly-rich loop might stabilize the bound ATP (Figure 5B). In the structure of the CagA-bound MARK2, the active conformation was induced by the presence of the CagA peptide, which mimics the binding of a typical substrate to the P+1 loop in the kinase domain; however, the P+1 loop in our MELK KD-UBA structure distinctly protrudes, which would obstruct substrate binding (Figure 5C). Therefore, the kinase domain of MELK appears to adopt a conformation that allows the binding of ATP⋅Mg^2+^ but not an exogenous substrate.

**Figure 6:**
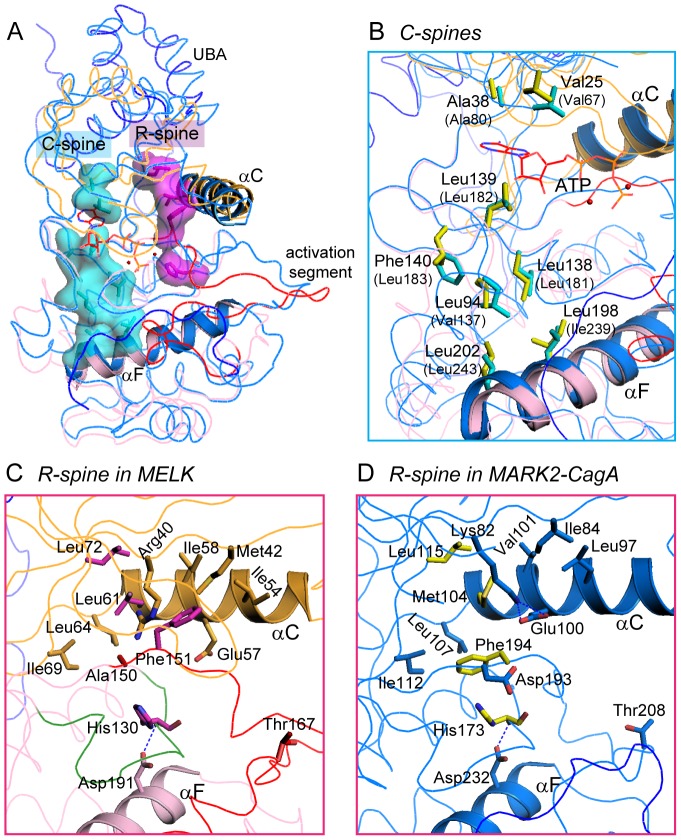
Comparison of the C-spine and R-spine in MELK and MARK2. (A) The C- and R-spines in our MELK KD-UBA structure. Residues in the C-spine and the R-spine of MELK are highlighted as cyan and magenta sticks, respectively, and additionally in surface model. The MARK2-CagA complex is shown for comparison, and the ATP molecule adapted from PKA (1ATP) is shown as red lines. The coloring schemes for MELK and MARK are as in Figure 5A. (B) Close-up view of the C-spine residues in MELK and MARK2-CagA. The corresponding residues of MARK2 are shown as yellow sticks and labeled in parentheses. (C and D) Close-up views of the R-spines of MELK (C) and MARK2 (D). Residues composing the R-spine and surrounding the DFG motif are shown as sticks. In panel C, the catalytic loop in MELK is colored green.

To further assess the catalytic ability of MELK, we then investigated two functionally important “spines” that are assembled or disassembled depending on the presence of ATP or substrate and are correlated with the activity of protein kinases [46], [47]. The two spines in the structures of our MELK KD-UBA and the active MARK2-CagA complex are generally superposed (Figure 6). The catalytic spine (C-spine) consists of eight highly conserved hydrophobic residues from both N- and C-lobes and is anchored to helix αF that is deeply buried in the C-lobe (Figure 6B). Residues Val25, Ala38 and Leu139 in MELK are optimally arranged and form a hydrophobic pocket to accommodate the adenine ring of a bound ATP molecule, as observed in the structures of the MARK2-CagA complex and other active kinases [42], [48]. The regulatory spine (R-spine) comprises four nonconsecutive hydrophobic residues from both lobes, which in MELK are Leu72 from strand β4, Leu61 from helix αC, Phe151 in the conserved DFG motif and His130 in the catalytic loop (Figure 6C). The R-spine is also connected to the buried αF helix via the hydrogen bond between His130 and Asp191 (Figure 6C). In the active MARK2-CagA structure, Phe194 in the DFG motif joins the two lobes through considerable hydrophobic interactions, and the adjacent Asp193 is appropriately positioned to ligate the potential ATP⋅Mg^2+^ (Figure 6D). Moreover, the highly conserved Lys82 and Glu100 form salt bridges, which are a hallmark of protein kinases in their activated state. In our MELK structure, three residues of the R-spine in MELK are in perfect configuration as observed in the active MARK2-CagA structure; however, Phe151 adopts a distinct conformation due to the mutation of the preceding Asp150 to Ala (Figure 6C). The aromatic ring of Phe151 rotates approximately 180° and inserts between Glu157 and Lys40 (here, mutated to Arg40). The dramatic movement of Phe151 impedes the formation of the characteristic salt bridges, and more importantly, the proper assembly of the active R-spine, resulting in the unphosphorylated, inactive MELK. Therefore, the C-spine of the unphosphorylated MELK is appropriately configured even in the absence of ATP⋅Mg^2+^, whereas the R-spine adopts a partially distorted conformation due to the K40R/D150A double mutation.

Most recently, the structure of a similar MELK fragment (residues 2–340) was reported (PDB ID: 4BL1, superseding 3ZGW). In this wildtype, unphosphorylated MELK structure, the activation segment is partially disordered (residues 157–170, including the key Thr167), whereas the traceable residues of the P+1 loop adopt a similar protruded conformation as observed in our unphosphorylated MELK mutant structure (Figure S5A). The reported structure contains a bound AMP-PNP molecule, and both its C-spine and R-spine exhibit typical active conformations (Figure S5B and C). Our structure of the unphosphorylated mutant MELK largely superposes to this reported structure of wildtype MELK, except for the aforementioned conformational deviations caused by the introduced mutations.

### The unique intramolecular disulfide bond within the activation segment

**Figure 7:**
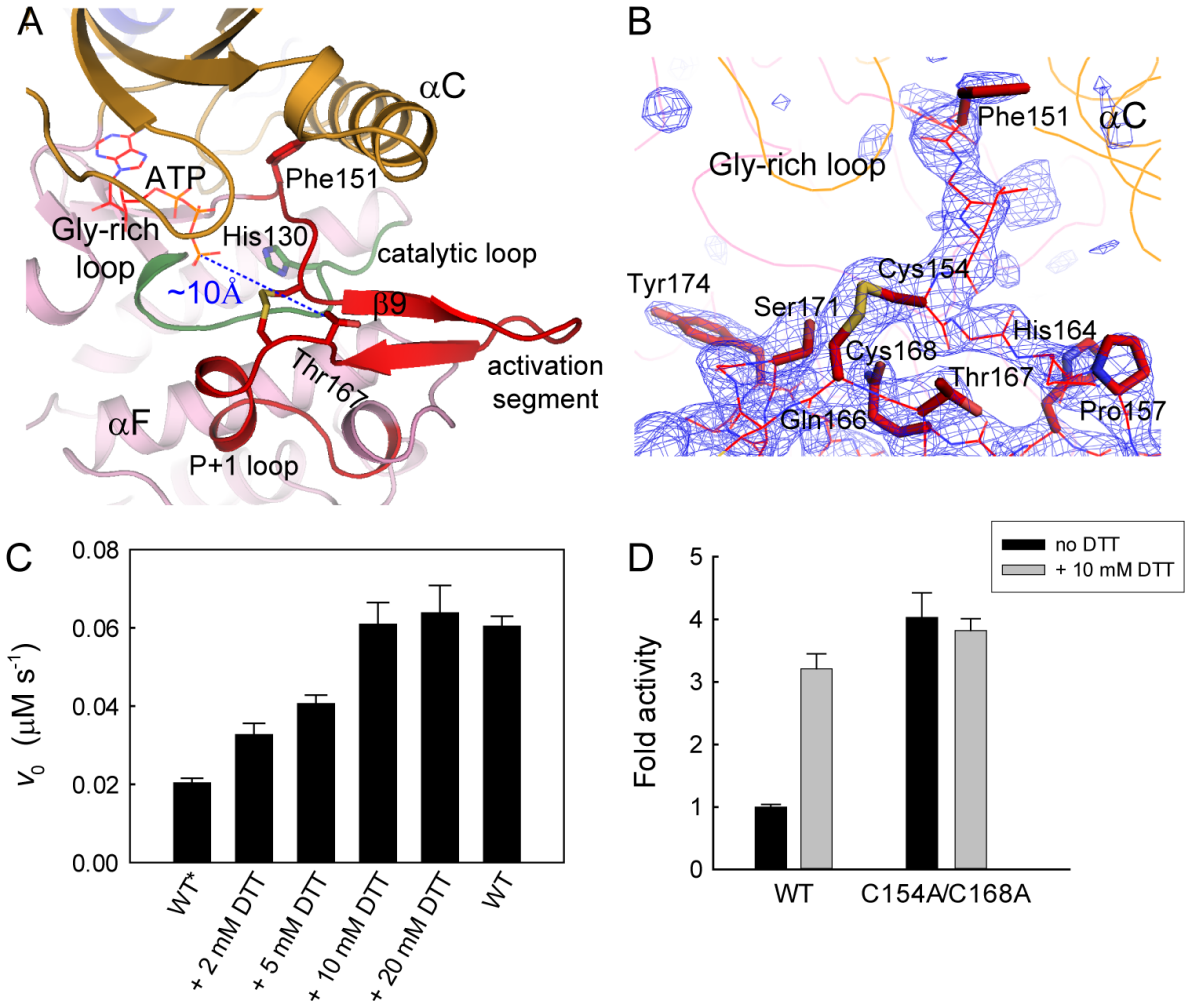
The intramolecular disulfide bond in the activation segment. (A) Ribbon representation of the activation segment of MELK. The molecule is colored as in Figure 2A, except that the catalytic loop is colored green. The intramolecular disulfide bond and certain key residues are highlighted as sticks. The ATP molecule shown as red lines was adapted from PKA (1ATP), and the distance between this potential ATP and Thr167 is indicated by a blue line. (B) The *F_o_ - F_c_* omit map (contoured at 3.0 σ) clearly shows electron density for the disulfide bond formed between Cys154 and Cys168. Residues in the activation segment are shown as red lines, and the side chains for Phe151 in the DFG motif, the critical Thr167 and several other residues are highlighted as sticks. (C) Effect of DTT on MELK activity. The wildtype MELK KD-UBA was purified in the absence (WT*) or presence (WT) of 5 mM DTT. The assays were performed in standard kinase assay buffer, in the presence of 1 mM ATP, 10 µM AMARA, 100 nM MELK (WT* or WT) and the indicated concentrations of DTT (mean and s.e.m., n = 3). (D) The activity of the cysteine mutant is DTT independent.

The activation segment in our MELK structure adopts a conformation that is different from either the inactive or the active MARKs (Figure 5). On close inspection, we unexpectedly discovered two disulfide bonds involving three Cys residues within the activation segment of MELK (Figure 1A). One disulfide bond is intramolecular and formed between Cys154 and Cys168 (Figure 7A and B), and the other is intermolecular and formed between two Cys169 residues from symmetry-related molecules (Figure S6). Cys169 is highly conserved in AMPK-related kinases, and such an intermolecular disulfide bond, which is likely a crystallization artifact, has been found in other KD-UBA structures in different space groups [41], [43]. Conversely, the intramolecular disulfide bond connects the middle region of the activation segment in our structure and has not been reported in other AMPK-related kinases. An antiparallel β-sheet between strand β6 in the catalytic loop and β9 in the activation segment is characteristic of the active state of kinases; however, strand β9 in our MELK structure forms an antiparallel sheet with an additional β-strand that precedes the critical Thr167 and thus extends away from the catalytic loop (Figure 7A). Therefore, this intramolecular disulfide bond leads to the structural distortions that would impair the activity of MELK.

Although members of the AMPK-related kinase family are generally phosphorylated and activated by upstream kinases, MELK can be regulated through autophosphorylation of the critical Thr167 in the activation segment. Interestingly, the autophosphorylation of MELK and its kinase activity towards an exogenous substrate were both reported to completely depend on the presence of reducing agents [20]. We then purified the wildtype MELK KD-UBA protein in the absence of DTT and examined its kinase activity in the presence of different concentrations of DTT (Figure 7C). The activity of MELK towards the AMARA peptide as an exogenous substrate clearly demonstrated dependence on the DTT concentration, as reported towards the protein substrate MBP [20]. Double mutation of Cys154 and Cys168 abolished the dependence of MELK activity on reducing agents (Figure 7D). Therefore, our structure reveals the molecular mechanism of the dependence of MELK activity on reducing agents, and the biochemical analyses demonstrate that the elimination of the intramolecular disulfide bond is required for the full activation of MELK.

### Functional implication

The presence of a UBA domain is a prominent feature of the AMPK-related kinase family. Here, we report the structure of the KD-UBA fragment from a third family member, MELK. The UBA domain in MELK adopts the noncanonical UBA fold as that in the reported MARK-UBA and AMPK-AID structures [25], [27], [29], [41]–[43]. The MELK-UBA and MARK-UBA domains bind solely to the N-lobe of the kinase domain, whereas the AMPK-AID binds to both N- and C-lobes (Figure 2). Nevertheless, these interactions all involve the important αC helix, the conformation of which is a signal for the active and inactive states of most kinases. In both our and the reported (4BL1) MELK KD-UBA structures, the UBA domain binds more tightly to its kinase domain than that in MARKs (Figures 3 and S2). The mutation of the interface residues resulted in some constructs that were completely insoluble or partially soluble, and all of the soluble mutants exhibited impaired activities (Figure 4). Our structural and biochemical analyses suggest that the UBA domain is indispensable for MELK function, which may relate to the possible role of the MELK-UBA in the assistance of the appropriate protein folding and its ability to possibly confine the active conformation of the kinase domain via a tight interaction with the N-lobe.

MELK is unique among the AMPK-related kinases because it undergoes autophosphorylation. However, the phosphorylatable Thr167 is approximately 10 Å away from the γ-phosphate group of a potential ATP molecule that would be deeply bound in the catalytic cleft (Figure 7A). To achieve the intramolecular phosphotransfer during autophosphorylation, Thr167 must be placed in proximity to the catalytic Asp132, together with the binding of ATP to the C-spine and the functional assembly of the R-spine. The assembly of the R-spine largely depends on the conformation of the activation segment. In our unphosphorylated MELK structure, the conformation of the activation loop is restricted by an intramolecular disulfide bond between Cys154 and Cys168 and is further stabilized by the presence of two antiparallel β-strands. Under reducing conditions, this disulfide bond would be broken, and the activation segment would be more flexible and able to undergo conformational changes. Subsequently, Thr167 could be presented to the catalytic Asp132 and the bound ATP and thereby poised for autophosphorylation. In summary, these structural analyses suggest that the unphosphorylated MELK possesses the ability to autophosphorylate its conserved threonine site because both the C-spine and R-spine are well assembled in the wildtype, unphosphorylated structure (4BL1) and because the intramolecular disulfide bond observed in our mutant structure would be broken by reducing agents.

In addition, the activity of MELK towards exogenous substrates also depends on reducing agents (Figure 7) [20], which might also be due to the presence of this unique intramolecular disulfide bond within the activation segment. When this disulfide bond is broken and Thr167 is autophosphorylated, the P+1 loop in the activation segment would concomitantly change to an active conformation that is capable of phosphorylating exogenous substrates. The kinase activity of MELK was reported to be required for the interaction with and subsequent activation of ASK1, and this function of MELK was increased by H_2_O_2_ [13]. MELK was found to be expressed at a significantly high level in a great majority of cancers [14], [15], and cancer cells are generally under oxidative stress, an imbalance of pro-oxidants and antioxidants. Reduction/oxidation, in addition to phosphorylation, might also serve as a regulatory mechanism for MELK activity (autophosphorylation/activation and phosphorylation of exogenous substrates) and thereby play an important role in MELK-associated cancer metastasis. Therefore, our structural and biochemical results reveal the roles of the UBA domain and reducing agents in regulating MELK activity, which provide the basis for developing specific inhibitors of MELK, particularly irreversible covalent inhibitors that target the essential cysteine residues.

## Supporting Information

Figure S1 **LC-MS analysis of the MELK KD-UBA fragment overexpressed in** ***E. coli*** **.**
(TIF)

Figure S2 **Comparison of the KD-UBA fragment from MELK and MARK.**
(TIF)

Figure S3 **Sequence alignment of the kinase domains from hMELK, mMELK, xMELK, zMELK, cePIG1 and hMARK1-4.**
(TIF)

Figure S4 **Circular dichroism (CD) spectra for MELK wildtype and two representative mutants.**
(TIF)

Figure S5 **The C- and R-spines in the reported MELK structure (4BL1).**
(TIF)

Figure S6 **Electron density map of the intermolecular disulfide bond.**
(TIF)

File S1 Supporting Figure legends for Figures S1, S2, S3, S4, S5, S6 and Supporting Methods S1.
(DOC)
