# Supplementary figures and images for: Structural Basis for the Regulation of Maternal Embryonic Leucine Zipper Kinase
Source: PLoS One. 2013 Jul 26;8(7):e70031. doi: 10.1371/journal.pone.0070031 (PMC3724675; doi:10.1371/journal.pone.0070031)

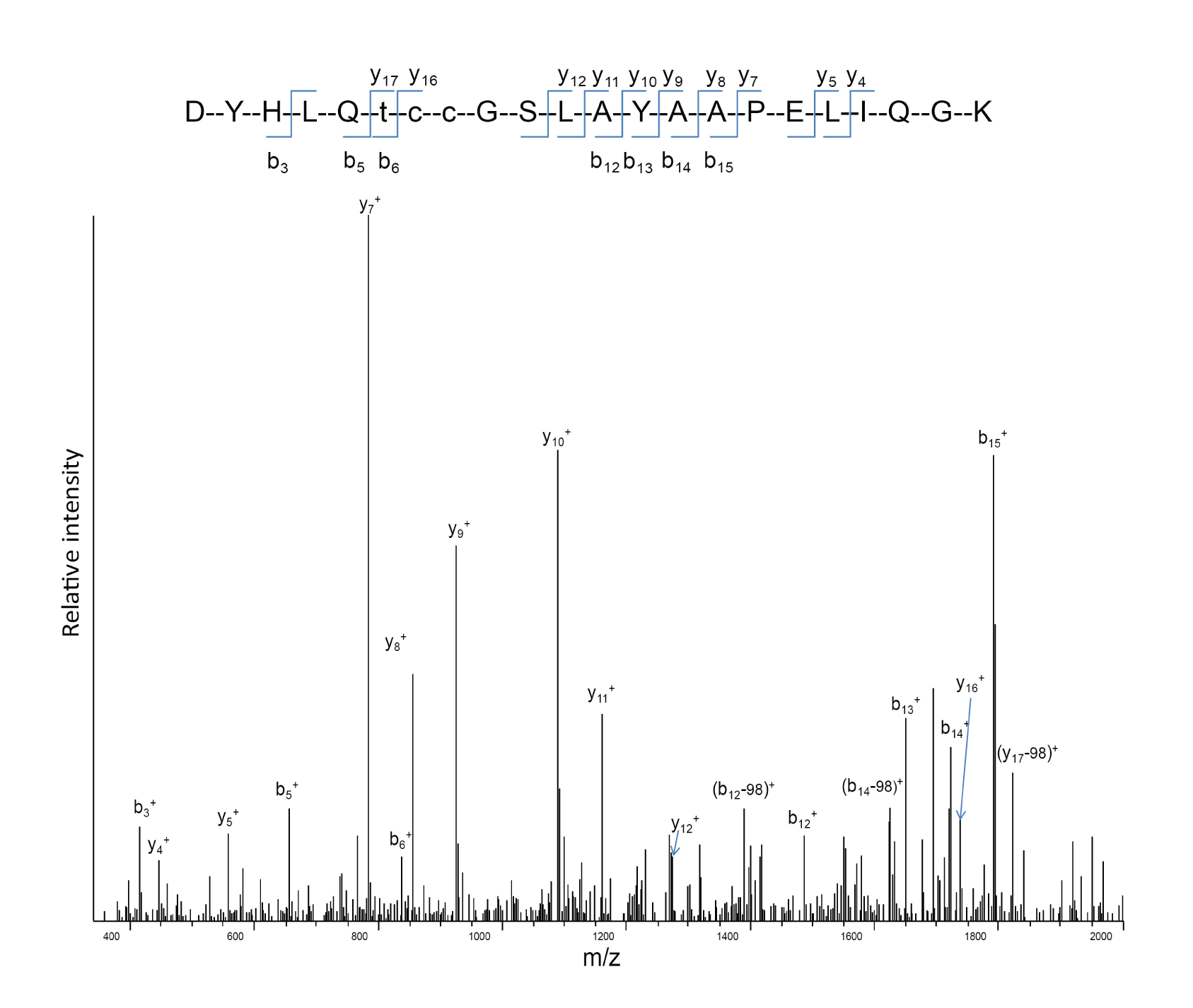

Supplement: Figure S1 — LC-MS analysis of the MELK KD-UBA fragment overexpressed in E. coli . (TIF) [file pone.0070031.s001.tif]

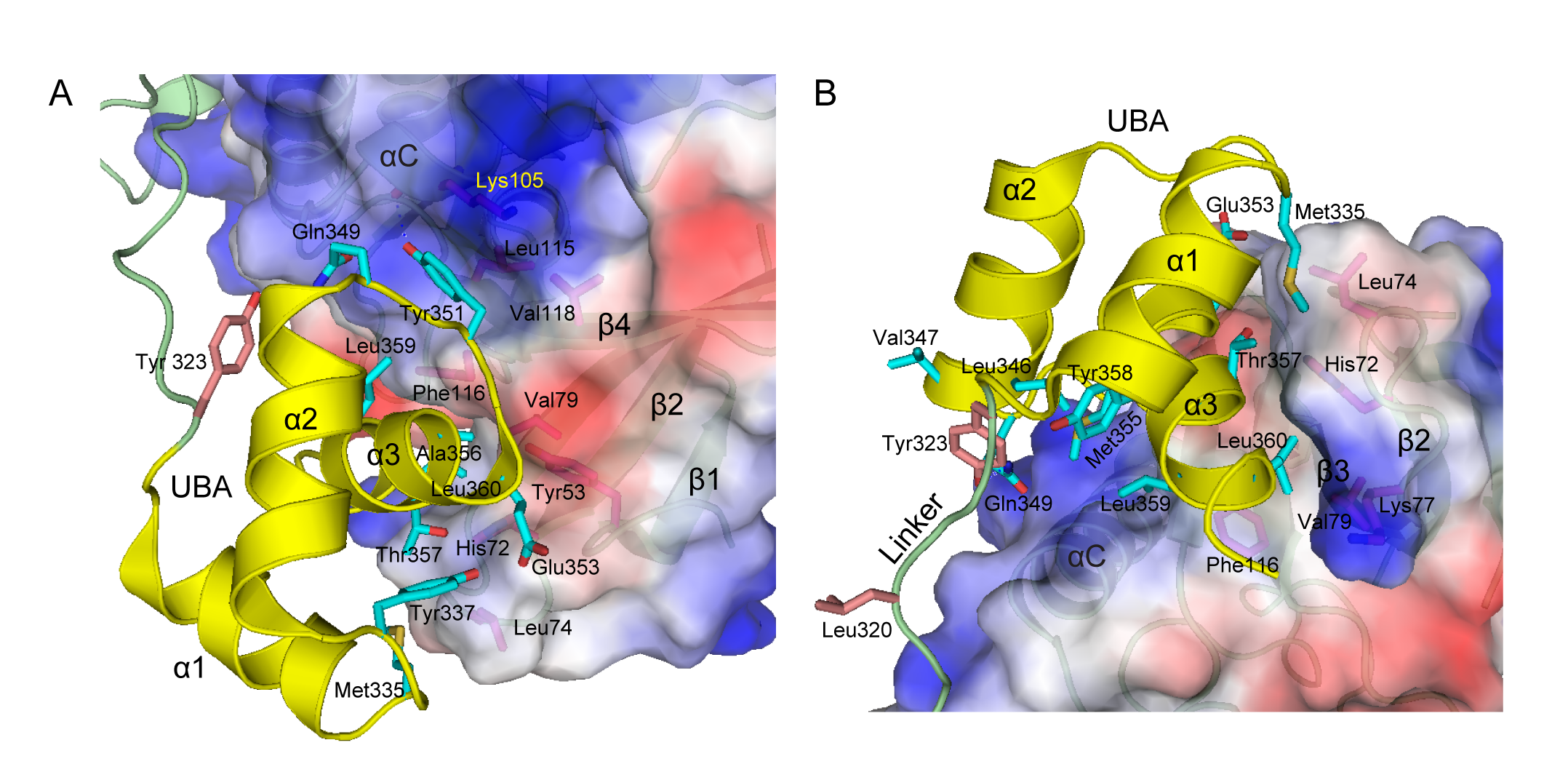

Supplement: Figure S2 — Comparison of the KD-UBA fragment from MELK and MARK. (TIF) [file pone.0070031.s002.tif]

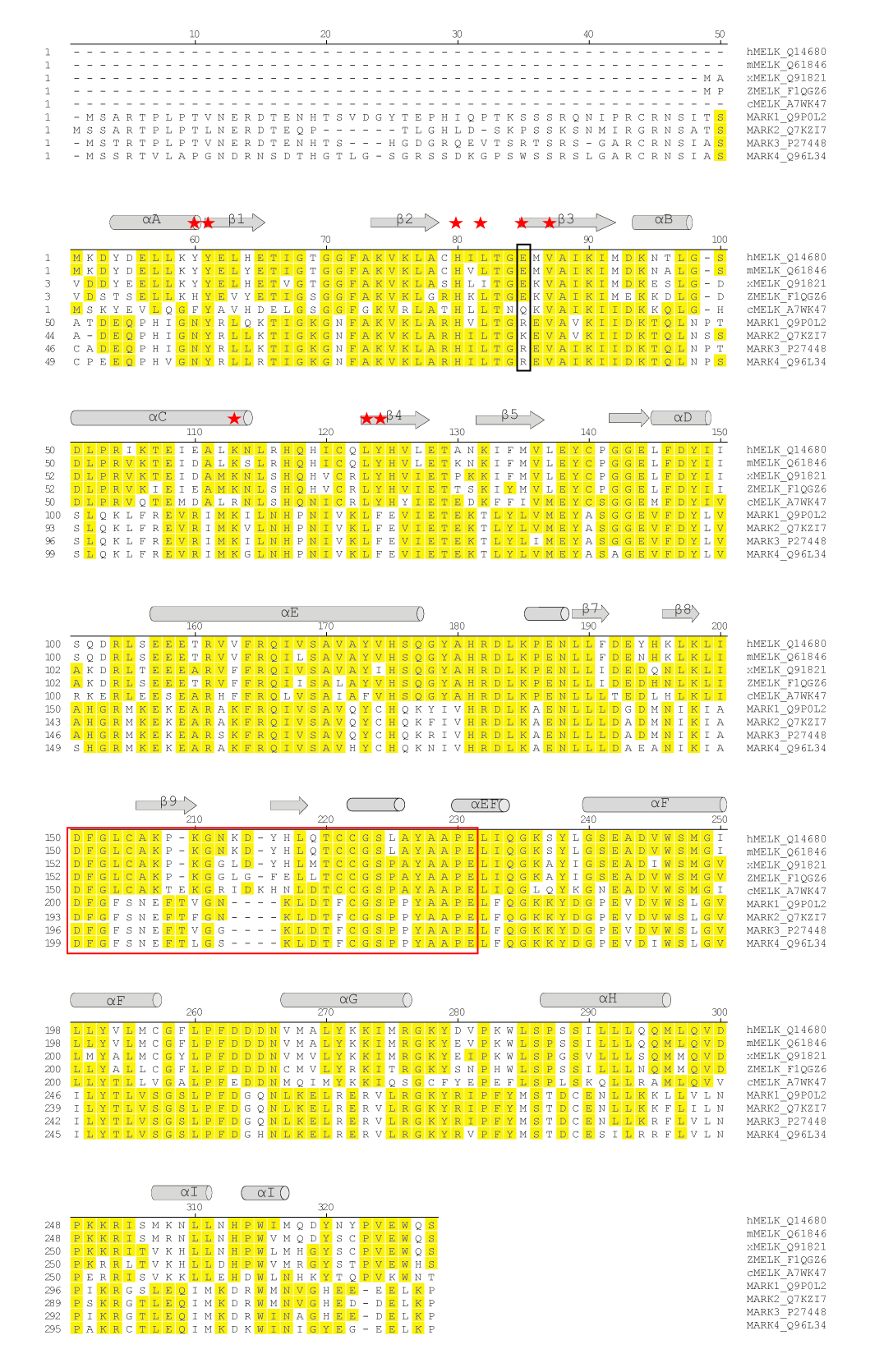

Supplement: Figure S3 — Sequence alignment of the kinase domains from hMELK, mMELK, xMELK, zMELK, cePIG1 and hMARK1-4. (TIF) [file pone.0070031.s003.tif]

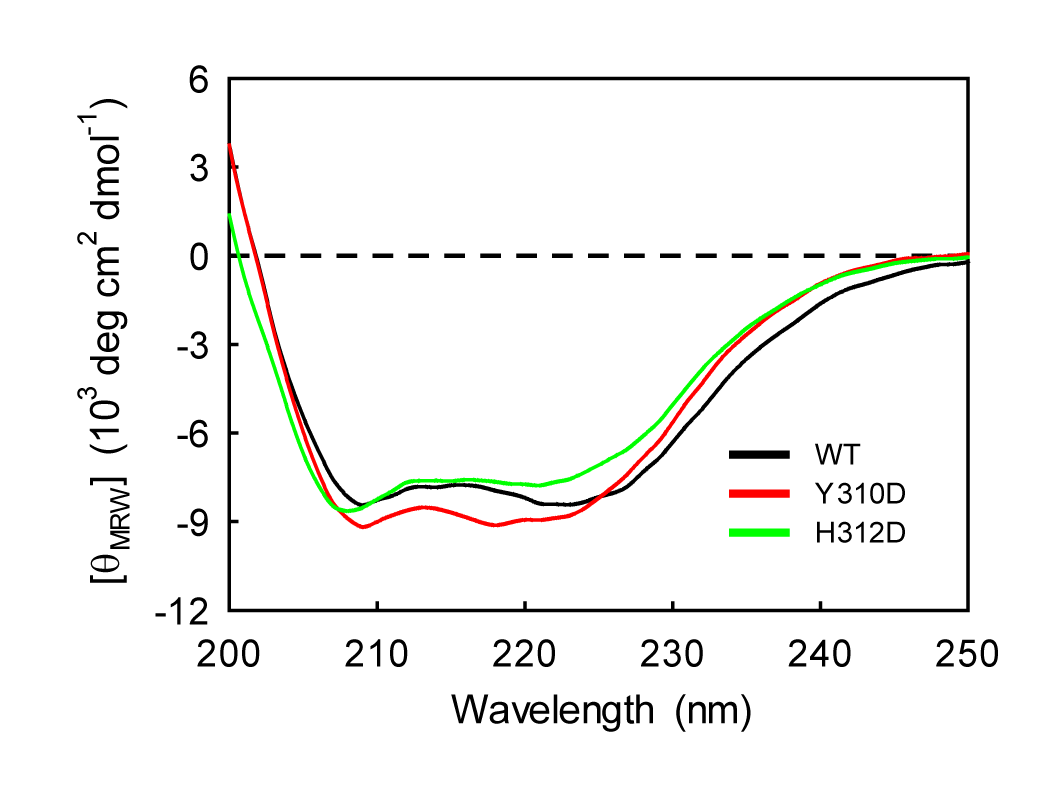

Supplement: Figure S4 — Circular dichroism (CD) spectra for MELK wildtype and two representative mutants. (TIF) [file pone.0070031.s004.tif]

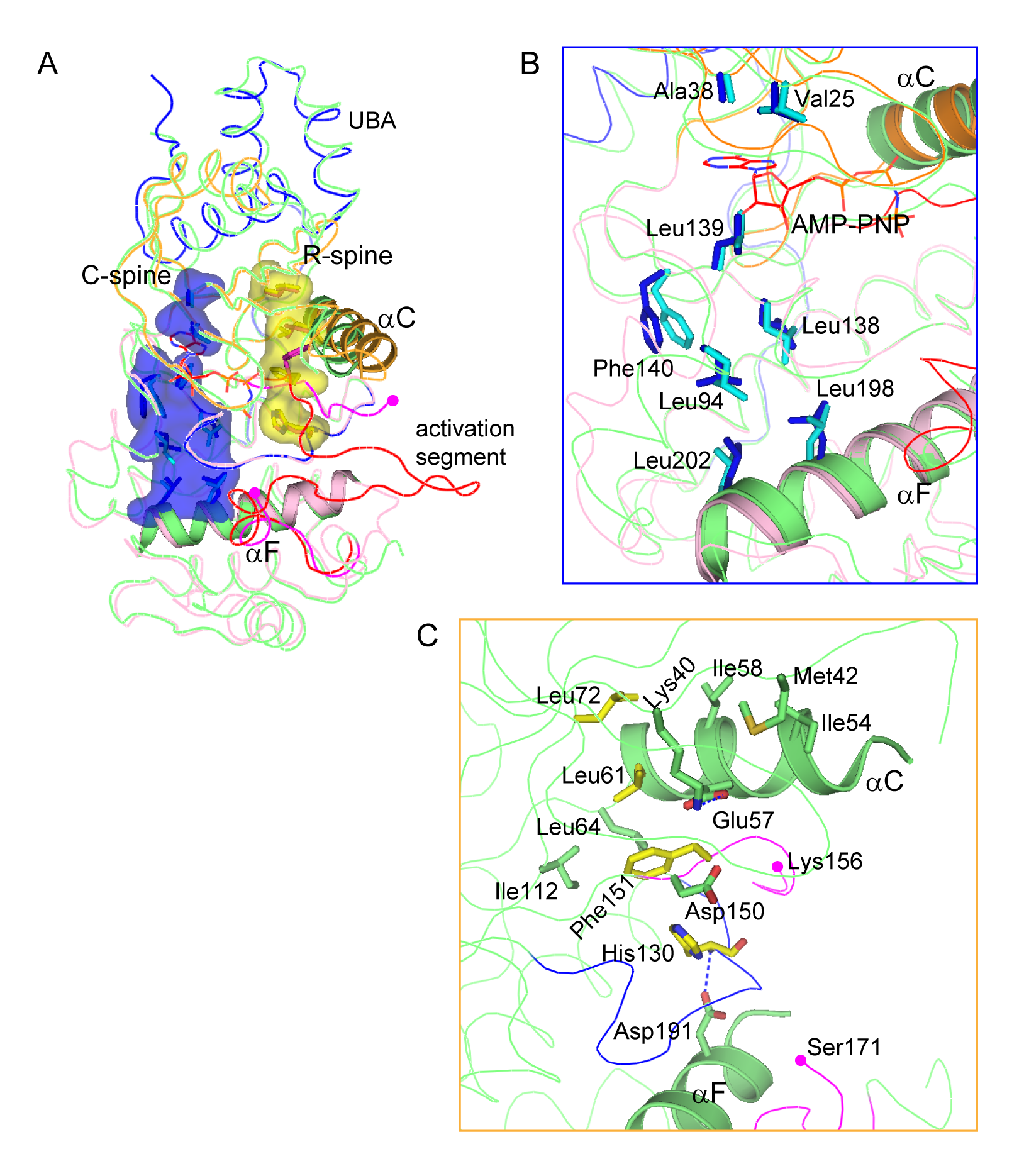

Supplement: Figure S5 — The C- and R-spines in the reported MELK structure (4BL1). (TIF) [file pone.0070031.s005.tif]

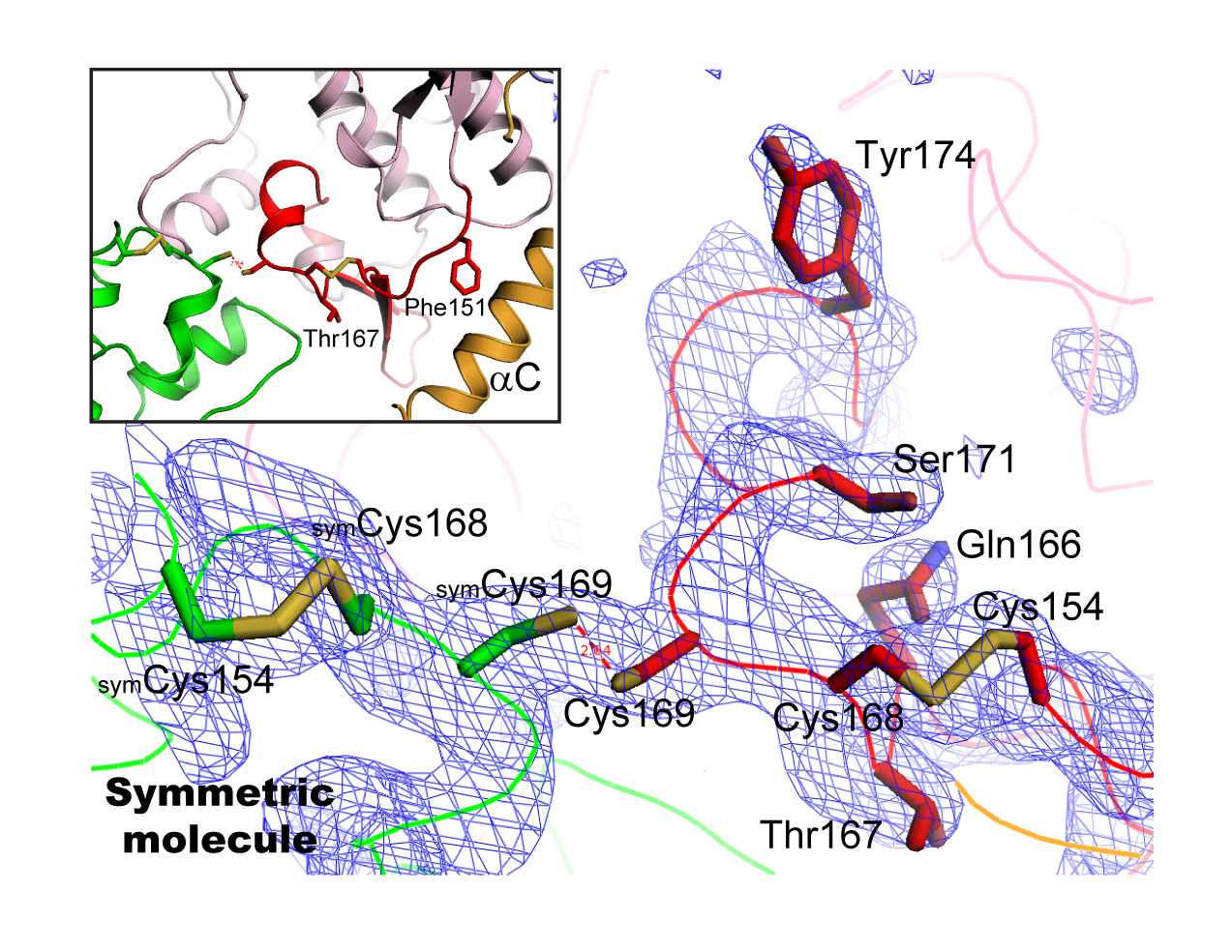

Supplement: Figure S6 — Electron density map of the intermolecular disulfide bond. (TIF) [file pone.0070031.s006.tif]
