# Supplementary material for: Structural Basis for the Regulation of Maternal Embryonic Leucine Zipper Kinase
Source: PLoS One. 2013 Jul 26;8(7):e70031. doi: 10.1371/journal.pone.0070031 (PMC3724675; doi:10.1371/journal.pone.0070031)
Supplement: File S1 — Supporting Figure legends for Figures S1, S2, S3, S4, S5, S6 and Supporting Methods S1. (DOC) [file pone.0070031.s007.doc]

**Supporting Information**

**Structural Basis for the Regulation of**

**Maternal Embryonic Leucine Zipper Kinase**

**Lu-Sha Cao^1^, Jue Wang^1^, Yuling Chen^1^, Haiteng Deng^1^, Zhi-Xin Wang^1^ and Jia-Wei Wu^1^***

^1^MOE Key Laboratory of Protein Sciences and Tsinghua-Peking Center for Life Sciences, School of Life Sciences, Tsinghua University, Beijing, China

*****E-mail: [jiaweiwu@mail.tsinghua.edu.cn](mailto:jiaweiwu@mail.tsinghua.edu.cn)

**Supporting Figures**

**Figure S1. LC-MS analysis of the MELK KD-UBA fragment overexpressed in *E. coli*.** Here shows the MS/MS spectrum of a double charged ion at m/z 1288.072 for MH_2_^2+^ corresponds to the mass of the mono-phosphorylated peptide DYHLQTCCGSLAYAAPFLIQGK. A loss of 98 Da (H_3_PO_4_) was observed for the precursor ion under the higher energy collision dissociation (HCD). The labeled peaks correspond to masses of y and b ions of modified peptides. The b_5_ and the phosphorylated b_6_ suggest that Thr6 (corresponding to Thr167 in hMELK) is phosphorylated.

(TIF)

**Figure S2. Comparison of the KD-UBA fragments from MELK and MARK.** Interactions between the kinase domain and the UBA domain of MARK2 (PDB ID: 2WZJ). The kinase domain and UBA of MARK2 are colored as Figure 2B, and the two views of the KD-UBA interface are oriented the same as that in Figure 3A and B. The residues on the kinase domain and the UBA domain that participate in hydrophobic and hydrophilic interactions are shown as magenta and cyan sticks, respectively, and the residues in the linker is highlighted as salmon sticks. Hydrogen bonds are indicated by blue dashed lines. The predominantly hydrophobic interactions in MELK are largely conserved in MARKs. For instance, the conserved Tyr351 in the loop between helices α2 and α3 makes hydrophobic interactions with the methylene groups of Lys105 on helix αC and two hydrophobic residues (Leu115 and Val118) from strand β4 (panel A). The hydroxyl group of Tyr351 also forms hydrogen bonds with the main chain carbonyl group of Lys105; however, Tyr351 is not further stabilized by the adjacent Glu349 (Trp308 in MELK). In addition, the conserved Leu360 inserts into a hydrophobic cave formed by Tyr53, His72, Val 79 and Phe116 (panel B). However, the additional polar contacts observed in MELK KD-UBA structure are largely missing in MARK2. In particular, the acidic Glu353 in MARK2 replaces the His312 in MELK that penetrates into a hydrophobic pocket on the kinase N-lobe (panel A and Figure 3A), and Lys77 in MARK2 substitutes Glu35 in MELK that hydrogen bonds to two basic residues from the additional turns of the UBA α3 helix (panel B and Figure 3B). Moreover, the conserved Leu74 in MARK2 (Leu32 in MELK) makes hydrophobic contacts with residues solely from the UBA domain (Met335 and Tyr337), while this interface in MELK also involves the C-terminal loop (panel A and Figure 3A). As to the interactions involving the linker N-terminal to the UBA domain, only Tyr323 (Leu282 in MELK) interacts with residues from the UBA domain (Leu346, Val347 and Tyr358, and Gln349), whereas Leu320 (Phe279 in MELK) is facing the solvent (panel B and Figure 3B). Thus, the interaction between KD-UBA in MARK is weaker compared to that in MELK.

(TIF)

**Figure S3. Sequence alignment of the kinase domains from *h*MELK, *m*MELK, *x*MELK, *z*MELK, *ce*PIG1 and *h*MARK1-4.** The sequence alignment was generated by ClustalW. The code following each protein name is the corresponding Swiss-Prot ID. The secondary structural elements are indicated above the alignment, and the activation segment is boxed in red. The key residues at the KD-UBA interface of human MELK are indicated by red asterisks above the alignment. It is noteworthy that the acidic Glu35 in MELK kinase domain is replaced by a basic side chain of Arg or Lys in MARKs (boxed in black).

(TIF)

**Figure S4. Circular dichroism (CD) spectra for MELK wildtype and two representative mutants.**  The far-UV circular dichroism (CD) spectra were recorded using 1 mm length cells on a Pistar π-180 spectrometer (Applied Photophysics Ltd). Protein samples were analyzed at a concentration of 2 μM in 5 mM Tris (pH 8.0) at room temperature. Data were collected over a wavelength range of 200-250 nm at 1 nm intervals; measurements were averaged for 3 scans. The CD spectra for wildtype MELK KD-UBA and two mutants are all profiles typical of predominantly α-helical structure with double minima at 208 nm and 222 nm, suggesting that the soluble MELK mutants display little conformational change compared to the wildtype protein.

(TIF)

**Figure S5. The C- and R-spines in the reported MELK structure (PDB ID: 4BL1)** (A) Comparison of our MELK KD-UBA structure with the reported structure. The reported structure is colored light green, and the AMP-PNP molecule is shown in red lines. Residues constituting the C-spine and R-spine in the reported MELK structure are highlighted as blue and yellow sticks, respectively, and additionally in surface model. Our MELK KD-UBA structure is colored as in Figure 2A, and the corresponding residues in the C- and R-spines are shown as cyan and magenta sticks, respectively. The αC and αF helices in both structures are highlighted. The activation segment and the catalytic loop in the reported structure are highlighted in magenta and blue, respectively. Notably, the traceable residues succeeding the DFG motif (corresponding to strand β9) interact with strand β6 in the catalytic loop, indicating an active-like conformation. However, the C-terminal part of the activation segment, including the traceable portion of the P+1 loop and the succeeding APE motif, adopts similar conformation as observed in our unphosphorylated MELK structure, preventing the binding of an exogenous substrate. (B) Close-up view of the C-spines in our MELK KD-UBA and the reported structures. The reported structure was generated with the wildtype, unphosphorylated MELK protein, and its C-spine was completed with the presence of the AMPPNP molecule. All eight residues of the C-spine in our structure are optimally arranged as observed in the reported structure. (C) Close-up view of the R-spine in the reported structure. The activation segment in the reported structure is colored in magenta, and the catalytic loop containing the important His130 is colored in blue. The central region of the activation segment (residues 157-170), including the key Thr167, was untraceable. The characteristic salt bridges between Lys40 and Glu57 and the hydrogen bond between His130 and Asp191 are indicated as blue dashed lines. Although the activation loop is partially disordered, the R-spine in this wildtype structure is well configured. Three R-spine residues Leu61, Leu72 and His130 in two MELK structures adopt the same position with the only exception of the Phe151 in the DFG motif. Because of the mutation of Asp150 to Ala, the aromatic ring of Phe151 in our mutant structure rotates approximately 180°, dramatically distinct from the traditional DFG-in conformation as observed in the wildtype structure.

(TIF)

**Figure S6. Electron density map of the intermolecular disulfide.** The original MELK molecule is colored as in Figure 2A, while the symmetric molecule is colored green. The *F_o_-F_c_* omit map (countered at 3.0 σ) for the region of two intramolecular and one intermolecular disulfide bonds is shown in blue. The inset is the cartoon representation at the same orientation, with the important Phe151 and Thr167 highlighted as sticks. The distance between two sulfur atoms of the symmetry-related Cys169 residues is 2.04 Å, which is typical for a disulfide bond.

(TIF)

**Supporting Method**

**Methods S1. LC-MS/MS analysis.** The bacterially expressed MELK KD-UBA protein were subjected to SDS-PAGE, and the corresponding gel bands were excised from the gel, reduced with 25 mM of DTT and alkylated with 55 mM iodoacetamide. In gel digestion was carried out with the sequence grade modified trypsin (Promega, Fitchburg, WI) in 50 mM ammonium bicarbonate at 37 °C, overnight. The peptides were extracted twice with 1% trifluoroacetic acid in 50% acetonitrile aqueous solution for 30 min. The extractions were centrifuged in a speedvac to reduce the volume, and then separated by a 65 min gradient elution at a flow rate of 0.25 µL/min with the EASY-nLCII™ integrated nano-HPLC system (Proxeon, Denmark) that interfaces directly with the Thermo LTQ-Orbitrap mass spectrometer. The analytical column was a home-made fused silica capillary column (75 µm ID, 150 mm length; Upchurch, Oak Harbor, WA) packed with C-18 resin (300 A, 5 µm, Varian, Lexington, MA). Mobile phase A consisted of 0.1% formic acid, and mobile phase B consisted of 100% acetonitrile and 0.1% formic acid. The LTQ-Orbitrap mass spectrometer was operated in the data-dependent acquisition mode using the Xcalibur 2.0.7 software and there is a single full-scan mass spectrum in the Orbitrap (400-1800 m/z, 30,000 resolution) followed by 20 data-dependent MS/MS scans in the ion trap at 35% normalized collision energy (CID) or 3 MS/MS scans using the electron transfer dissociation (ETD). The MS/MS spectra from each LC-MS/MS run were searched against the selected database using an in-house Mascot or Proteome Discovery searching algorithm.
